# Local Symmetry Deviation from the Average Structure of MnAs Revealed by Pair Distribution Function

**DOI:** 10.1021/acs.inorgchem.4c02667

**Published:** 2024-08-08

**Authors:** Dipankar Saha, Wojciech A. Sławiński, Susmit Kumar, Helmer Fjellvåg

**Affiliations:** † Center for Materials Science and Nanotechnology, Department of Chemistry, University of Oslo, P.O. Box 1033, Blindern, N-0315 Oslo, Norway; ‡ Faculty of Chemistry, University of Warsaw, Ludwika Pasteura 1, 02-093 Warsaw, Poland

## Abstract

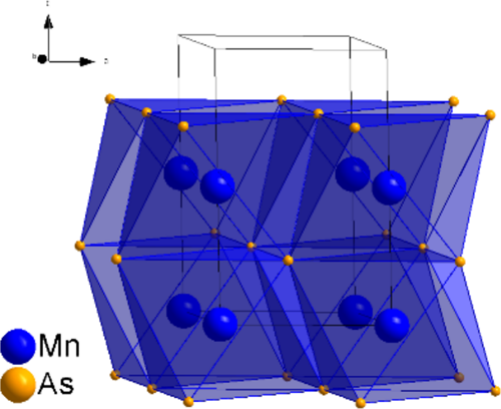

MnAs is an interesting material due to its magnetocaloric properties, which can be utilized in magnetic refrigeration. However, despite major efforts, its magnetic refrigeration performances in the substituted forms could not be improved compared to the parent MnAs phase. Both small and big box modeling of the pair distribution function of MnAs for the local structure description and powder X-ray diffraction for the average structure reveal an inherent local orthorhombic distortion in the hexagonal structure of MnAs. As a result of this distortion, any modification to the hexagonal structure results in an orthorhombic structure and a weaker magnetocaloric performance. This study highlights the importance of studying local distortion in magnetic materials. This is achieved by combining X-ray absorption spectroscopy with total scattering X-ray diffraction.

## Abstract 2

Due to the presence of inherent local distortion in MnAs revealed by modeling of pair distribution function, any modification to the structure leads to the formation of low-symmetry orthorhombic structure from hexagonal, resulting in low magnetocaloric efficiency.

## Introduction

Fundamental insight into structure–property relations is essential as the basis for optimizing materials for a sustainable future. However, if we seek to achieve such materials by iterative improvements to pre-existing approaches, we may not reach the advances that are required. To make a step-change in material performance, we need to understand underpinning relations across all length scales. Although conventional X-ray crystallographic methods provide an accurate description of the average structure of materials, they fail to address local symmetry deviations that could be responsible for emerging properties. It is important to note that the same average picture, as obtained from the Bragg diffraction, can be realized as an average of many different local atomic arrangements. The emerging technique of total scattering (TS)^1^ is capable of providing structural insight at local and average length scales. The Fourier transformation of TS data results in the pair distribution function (PDF) as a tool for the structural description of materials. In this article, we demonstrate how both small and big box modeling of atomic PDF data of MnAs can shine new light on its structure–property correlations.

MnAs is found as an extremely interesting material for applications in magnetoelectronics and spintronics. It can be grown epitaxially on GaAs and Si in terms of heterostructures for use in spin-polarized injection due to clean and sharp interfaces.^2−6^ There is currently a renewed interest in MnAs for energy-efficient refrigeration since it exhibits a giant magnetocaloric effect (MCE) under an external magnetic field of 5 T.^7^ The MCE can be utilized for refrigeration at room temperature and has the potential to replace the current vapor-compression technology,^8^ which suffers from drawbacks in terms of ozone-depleting refrigerants, toxicity, noise, and high energy consumption.

At ambient temperature and pressure, MnAs is a ferromagnetic metal (μ_F_/Mn = 3.4 μ_B_) with the hexagonal NiAs-type structure (α-phase, space group *P*6_3_/*mmc*). MnAs undergoes at *T*_C_ = 318 K a first-order magnetostructural phase transition to a paramagnetic (PM) orthorhombic MnP-type structure (β-phase, space group *Pnma*). The first-order magnetostructural transition (FOMT) results in a large entropy change that originates from the abrupt loss of magnetic long-range order. The phase transition is accompanied by a sharp decrease in the ordered magnetic moment and to some extent also in lowering of the Mn spin state, along with a discontinuous increase in resistivity and a discontinuous volume change (2.1%).^9,10^ A latent heat of 7490 J/kg is associated with the transition.^11^ The crystal structure reverts to the hexagonal NiAs-type phase (γ-phase) at *T*_t_ = 393 K via a second-order displacive phase transition. This high-temperature phase remains paramagnetic and metallic, with manganese atoms formally in a high-spin state. The stabilization of the γ phase is attributed to enlarged volume owing to thermal expansion.

The FOMT at 318 K is responsible for the giant MCE, magnetoresistance, and magnetoelastic effects.^9,12^ The entropy change (Δ*S*) reaches 40 J/(kg·K) at ambient pressure and increases further upon compression.^13^ The high-spin configuration for the ordered low-temperature structure is crucial for such a high entropy change. For low substitution levels (<3 atom %) at the Mn-site with transition metals M = Ti, V, Cr, Fe, Co, Mo, *T*_C_ typically decreases significantly while the FOMT character remains. At higher substitution levels, the orthorhombic structure (β phase) is stabilized to low temperature, the spin state of Mn is lowered, an incommensurate double spiral structure emerges, and the spin-disordering process has a weak first-order character.^14−17^ Similar observations are made in case of substitutions at the As site with P and in some respects also for Sb.^18−20^ These facts emphasize the connection between the magnetic and structural properties of MnAs. It was earlier predicted that the orthorhombic distortion would induce antiferromagnetic order and reduced crystal volume,^21,22^ later experimentally supported by the observation of an antiferromagnetic double spiral spin structure, both for substituted MnAs and for MnAs under external pressure. Bean and Rodbell proposed a model where the exchange interactions are sensitive to lattice strain.^11^ Building further on, their model recent density functional theory (DFT) studies predict that the exchange interactions do not only depend on volume but that the degree of the orthorhombic distortion of the structure also plays an important role.^23^ This demonstrates that lattice distortions and spin–lattice coupling play important roles in the phase transitions in MnAs and its derivative phases. In the current work, we address how atomic PDF data can shed light on local structure distortions in the ferromagnetically ordered low-temperature phase of MnAs at the verge of transitioning into the structurally related orthorhombic structure. The results are believed to be of general importance for similar transitions. To achieve our PDF conclusions, we apply both small and big box modeling.

## Experimental Section

MnAs was synthesized from the pure elements Mn and As (>99.97%) in evacuated sealed quartz tubes. Mn lumps were crushed into fine powder using an agate mortar and pestle, and stoichiometric amounts of Mn and As were loaded in an alumina crucible inside a quartz tube. The tube was evacuated slowly to avoid any powder loss. The tube was sealed under vacuum and placed in the isothermal zone of a standing furnace to avoid any sublimation of As. The tube was heated to 900 °C over a period of 1 week and cooled (1 °C/min) to room temperature. The resulting material was crushed thoroughly and reheated for 1 week at 900 °C (heating and cooling rates 1 °C/min) to obtain the final fully phase pure product, totally free from MnO or other impurities.

Sample purity and homogeneity were ascertained from powder X-ray diffraction (PXRD) data recorded using a Bruker D8 Discover diffractometer with the Bragg–Brentano geometry, Cu Kα1 radiation [λ = 1.540598 Å; Ge(111) monochromator], and a LynxEye detector. Room-temperature total scattering data were collected at the Materials Science Beamline^24^ at the Swiss Light Source (SLS; Paul Scherrer Institut, Villigen). Silicon was used as a calibrant for wavelength and instrumental parameters (*a*_Si_ = 5.431194 Å at 22.5 °C, NIST powder diffraction standard 640c). The diffractometer was operated in a Debye–Scherrer geometry with a Mythen microstrip detector and a capillary spinner. The wavelength was 0.406080 Å. The Mythen microstrip detector was positioned at four different 2Θ positions in order to collect a full set of angular data extending to high *Q*_max_ for the subsequent PDF analysis. The samples were packed under Ar atmosphere in a 0.3 mm diameter quartz capillary and sealed to avoid any oxygen/moisture in the capillary. The data collection time was 40 min. The diffraction data were normalized and reduced by standard routines at the beamline. The xPDFsuite^25,26^ was used for correction and Fourier transformation of the total scattering structure function *S*(*Q*) to obtain the PDF. *Q*_min_ = 0.5 Å^–1^ and *Q*_max_ = 28 Å^–1^ were used for the Fourier transformation. Experimental resolution parameters *Q*_damp_ = 0.0038 and *Q*_broad_ = 0.00229 were determined through refinements of PDF data for the Si standard. Unit cell parameters, anisotropic thermal factors, and δ2 and symmetry-allowed positions were refined to give the best fit to the experimental data. Variable-temperature PXRD data were collected at Swiss–Norwegian Beamline BM31, European Radiation Synchrotron Facility (ESRF), Grenoble, France.^27^ The wavelength of the X-rays was 0.27079 Å. The MnAs powder was packed in a 0.3 mm diameter quartz capillary inside a glovebox and sealed. A gas blower was used to heat the samples. The temperature at the sample was equilibrated for 5 min before each data collection.

Extended X-ray absorption fine structure (EXAFS) data were collected at the Mn K-edge (5900 eV) in transmission mode at the SuperXAS beamline at the Swiss Light Source synchrotron facility (Paul Scherrer Institut, Switzerland).^28^ MnAs was mixed and well ground with boron nitride (1:10 weight ratio). The powder was packed in a 0.5 mm quartz capillary in argon atmosphere to avoid any moisture, and data were collected at 25 °C. The analysis of the EXAFS spectra was performed with the IFEFFIT program package ATHENA ARTEMIS, HEPHAESTUS.^29^

## Results and Discussion

**Figure 1:**
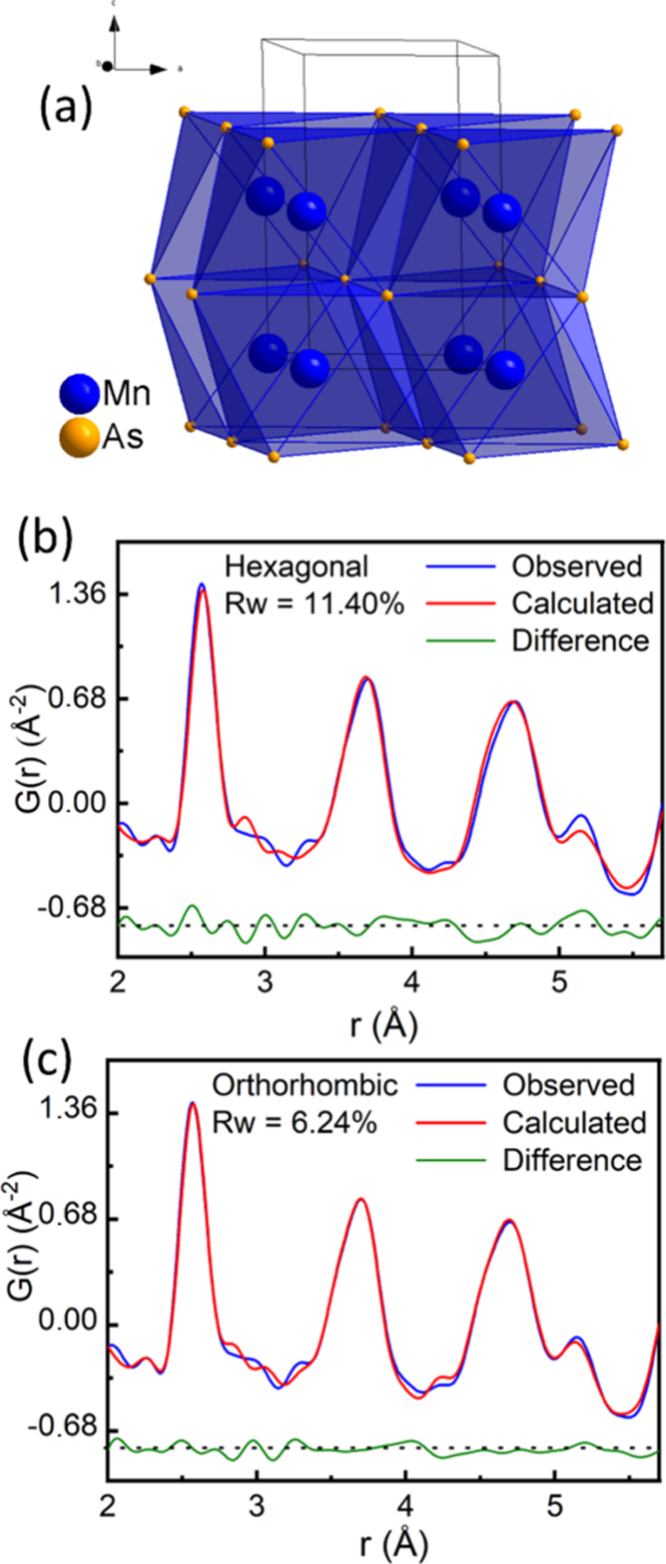
(a) Crystal structure of hexagonal MnAs (Mn: blue, As: yellow), (b) PDF data fitted with the hexagonal structure. (c) PDF data fitted with the orthorhombic structure in the *r*-range of 2–5.5 Å by using a small box approach.

The high-quality synchrotron data and the subsequent Rietveld analysis confirmed complete phase purity and the hexagonal crystal structure of MnAs at 298 K; *P*6_3_/*mmc*; *a* = 3.72132(3) Å, *c* = 5.70548(2) Å. Manganese is octahedrally coordinated by As atoms with a Mn–As bond distance of 2.579(1) Å. The MnAs_6_-octahedra share faces along [001] (see Figure 1a). The –Mn–Mn– atoms form straight one-dimensional (1D)-chains along [001] with an s bond distance of 2.852 Å, whereas there are six equivalent Mn–Mn bonds (3.721 Å) within the hexagonal ab-plane, in full agreement with previous data. However, a detailed analysis of the local- and the intermediate-range structures based on PDF data in real space reveals significant differences. The first peak at 2.539 Å corresponds to the first neighbor distances, i.e., of the MnAs_6_ octahedron, slightly shorter than the bond distance obtained from the average diffraction analysis, 2.579(1) Å. Note that slightly shorter Mn–As bond distances occur for the deformed octahedra in the orthorhombic (*Pnma*) variant of MnAs being stabilized on moderate metal atom substitution.^30^

In order to probe a possible local distortion, small box modeling of PDF was carried out at different *r*-ranges. The hexagonal model can be fitted for the *r*-range of 2–40 Å with *R*_w_ = 9.55%. When PDF refinements were restricted to the *r*-range of 2.0–5.5 Å, *R*_w_ increases to 11.40% (Figure 1b). For this *r*-range, the –Mn–Mn– chain along [001] is included in the length scale. On the contrary when using the orthorhombic model, the fitted *R*_w_ comes down to 6.24% (Figure 1c). A combined refinement of the orthorhombic structure for the *r*-range of 2.0–5.5 Å and a hexagonal structure for the *r*-range of 5.5–40.0 Å yields an *R*_w_ of 8.45%. These results indicate that there exists a local orthorhombic distortion that cancels out in the average structure with hexagonal symmetry. Analysis of the small box modeling suggests that the Mn–Mn–Mn bond angles in the chains deviate from the ideal value of 180° and is around 176.3° locally. When refining anisotropic displacement parameters for both Mn and As in Rietveld refinements (Table S3), we observe that the *U*_11_ and *U*_22_ [0.0076(3) Å^2^] parameters of Mn are clearly higher than those of *U*_33_(0.0026(3) Å^2^). This indicate thermal or static displacement of Mn atoms away from their ideal crystallographic positions within the hexagonal ab-plane. Similar anisotropic displacements were reported by Petkov et al. based on PDF and Rietveld refinements.^31^ The current PDF and Rietveld analyses show that the orthorhombic distortion in the MnP-type high-temperature phase is already indicated at the local level in the hexagonal average structure below the phase transition temperature. Furthermore, to analyze the local structure, Mn K-edge EXAFS spectra were analyzed. Both orthorhombic and hexagonal models were used to fit the EXAFS data for the first coordination shell of Mn. An improved fit to the orthorhombic structure model relative to the hexagonal structure indicates that the local structure might deviate from hexagonal symmetry (see Supporting Information Figure S6) A signature of such local disorder has been observed in earlier EXAFS studies. It was reported that the local disorder in the Mn–As distances in the basal plane of the hexagonal phase rather than between the Mn–Mn subshells is described below.^32^

**Figure 2:**
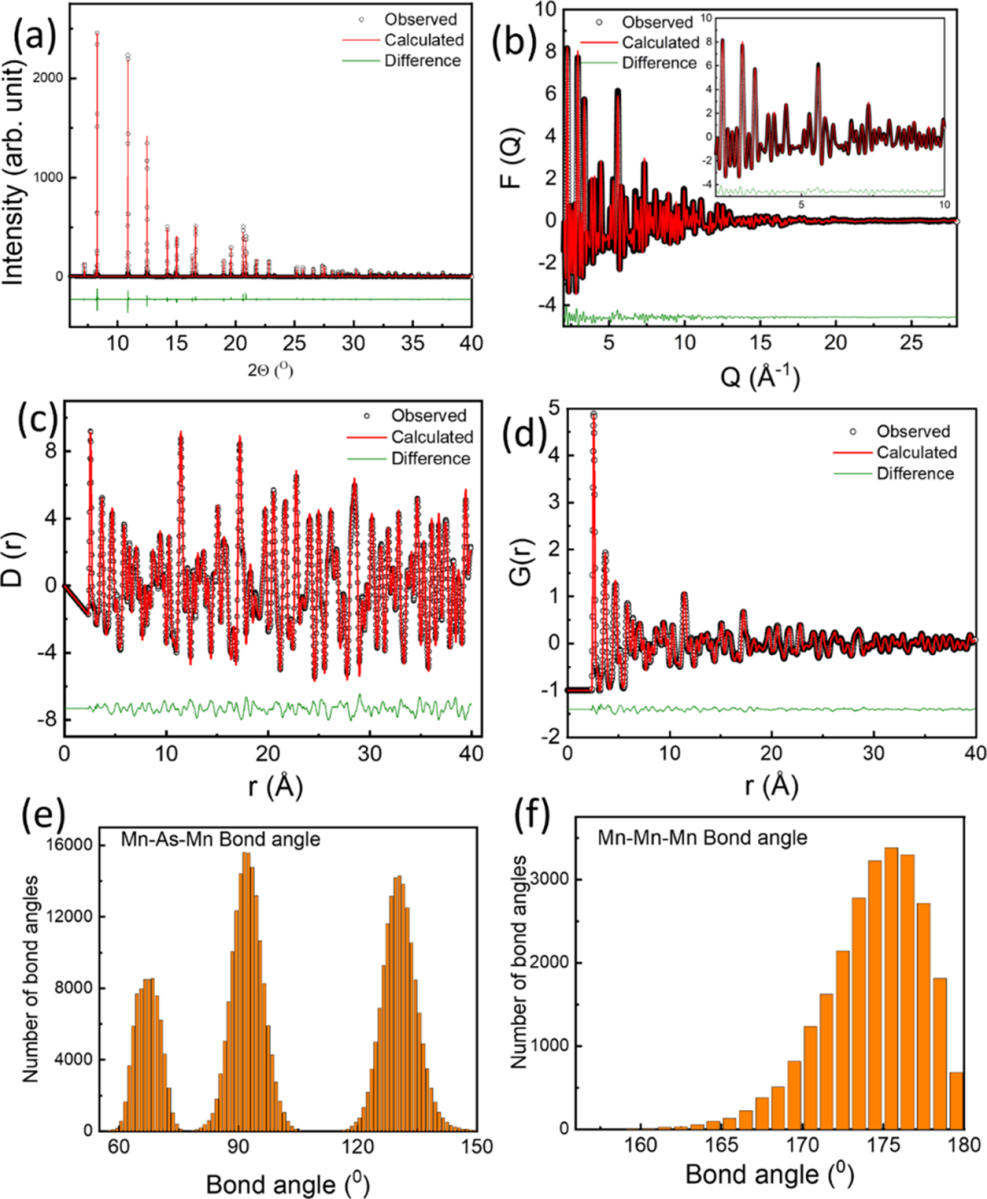
(a) Rietveld fit of the diffraction pattern, (b) *F*(*q*) (inset shows a zoomed view of the low *Q* region), (c) pair distribution function *D*(*r*), (d) radial distribution function *G*(*r*), (e) Mn–As–Mn bond angle distribution, and (f) Mn–Mn–Mn bond angle distribution.

The small box modeling of the PDF and EXAFS data indicates that local displacements are present in MnAs at 298 K. One challenge in the fitting is bias toward the chosen symmetry. In order to investigate the orthorhombic distortion and exclude symmetry bias imposed by a space group symmetry, big box modeling of the PDF data was carried out by means of the RMC (Reverse Monte Carlo) method and the RMCProfle7 package.^33,42^ The supercell consisted of 30 × 30 × 14 unit cells. Figure 2 shows the fitting of Bragg data (panel a), total scattering factor *F*(*Q*) (panel b), differential correlation function *D*(*r*) (panel c), and total radial distribution function *G*(*r*) (panel d). Partial functions contributing to the overall PDF are shown in Supporting Information Figure S3. The bond angle distributions for Mn–As–Mn and Mn–Mn–Mn are shown in Figure 2e,f, respectively. The Mn–As–Mn bond angles are centered around 67, 92, and 130° (values obtained from the Rietveld refinement of the average structure) within the error limit. However, the Mn–Mn–Mn bond angle distribution shows a peak at 176°, which deviates significantly from the average structure value of 180°. Both small box and big box modeling of PDF data suggest a similar deviation from the average picture. The orthorhombic structure (*Pnma*; MnP-type) has twice the volume of the hexagonal unit cell due to the symmetry lowering where atoms are systematically displaced out of their higher-symmetry points. The respective unit cells are related by **a**_O_ = **c**_H_; **b**_O_ = **b**_H_ and **c**_O_ = 2**b**_H_ + **a**_H_ (see Figure 1a for unit cell setting comparison). In slightly substituted MnAs (MnP-type MnAs_1–*x*_P*_x_* or Mn_1–*x*_M_x_As; M = V, Cr, Fe, Co, Ni, Mo), the Mn atoms forms zig-zag chains along **a**_**O**_ with Mn–Mn–Mn bond angles of 167° compared to 180° for the hexagonal structure. Our PDF data for hexagonal MnAs at 298 K suggest local distortions where Mn atoms are systematically shifted away from the –Mn–Mn–Mn– straight-chain configuration. This gives rise to orthorhombic distortions at short-range while maintaining an overall hexagonal symmetry for the long-range average structure.

**Figure 3:**
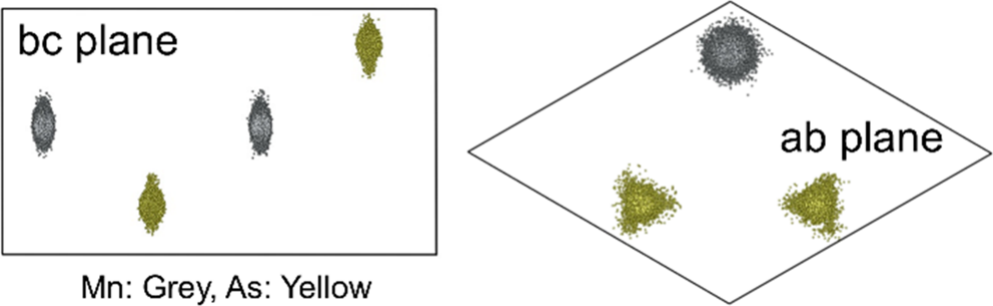
Unit cell projection of all atoms from big box RMC modeling.

Analysis of vectors describing the rotation and magnitude of the MnAs_6_ octahedral distortions (obtained from RMC refinement) shows that the octahedra are on average equally distorted in all three directions resulting in cancelation and thereby keeping the hexagonal symmetry intact (see Supporting Information Figure S6). The projection of the RMC modeled atoms into the unit cell (Figure 3) reveals that both Mn and As take an ellipsoidal distribution on the ab-plane instead of a spherical distribution. The deviation from spherical distribution and the inclination toward displacements in the hexagonal ab-plane are in line with the strong magnetocrystalline anisotropy in MnAs, with the *c*-axis being the hard axis of magnetization with strong Mn–Mn interactions and Mn-spins being located within the hexagonal ab-plane.^34^ However, aspects of chemical bonding for the –Mn–Mn– chains and strain owing to unusually short Mn–Mn bond distances are essential factors. Since the magnetoelastic coupling^35^ is stronger in the hexagonal plane than perpendicular, we believe that the strong magnetic interactions along with strain will locally cause a shift of atoms out of their ideal hexagonal symmetry points while the average arrangement remains hexagonal in nature. It is worth noting that previous DFT studies have concluded that hexagonal symmetry is favored by the ruling magnetic interactions.^36^ Regarding the chemical bonding and strain argument, we notice that a similar symmetry change, at local and average levels, occurs for MnAs_1–*x*_P_x_ (*x* = 0.06, 0.12, 0.18) due to a continuous low-spin to a high-spin magnetostructural phase transition and a substantial reduction in the unit cell volume, as well as in the orthorhombic *a*-axis, corresponding to [001] for hexagonal MnAs.^37^ It is interesting to note that such local structural fluctuations in magnetite have also been attributed to coemergence of magnetic order below the Curie transition.^38^

**Figure 4:**
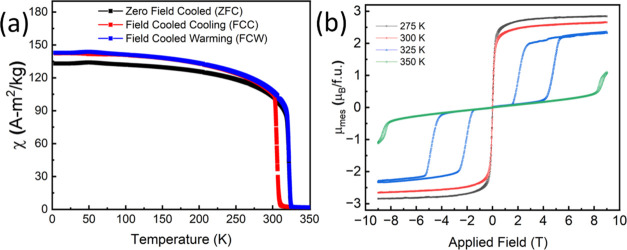
(a) Temperature-dependent ZFC, FCC, and FCW magnetization measured at 1 T for MnAs. (b) Isothermal magnetization of MnAs at 275 K (black square), 300 K (red circle), 325 K (blue triangle), and 350 K (green diamond).

The *H*–*T* magnetic phase diagram (Figure 4) for MnAs reveals that the hysteresis of the FOMT is maintained up to the second-order phase transition at around 393 K.^39^ The current magnetization data show how this hysteresis region connected with ferromagnetic MnAs appears in the *M*(*H*) data at 325 and 350 K (Figure 4). Probably, this process starts with the formation of nanodomains of high-spin MnAs with hexagonal symmetry within a matrix of lower-spin orthorhombic MnAs. An interesting question is whether such a process also occurs on heating, at *H* = 0, i.e., that nanoscale hexagonal islands form before the collective transition from MnP- to NiAs-type. Correspondingly, can the formation of nanoscale orthorhombic islands be a precursor to the FOMT and therefore responsible for the reduction in *X*(*T*) prior to the FOMT, or does the entire structure slightly deformed locally as discussed based on the PDF findings above?

**Figure 5:**
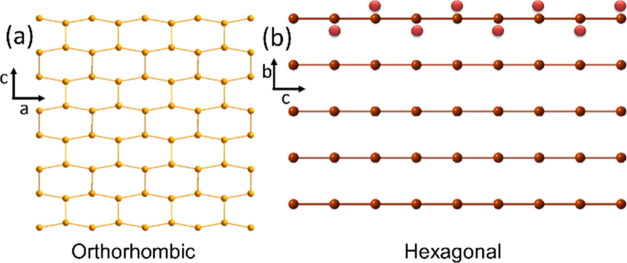
(a) Zig-zag chain formation in orthorhombic MnAs. (b) Linear Mn–Mn chains in hexagonal MnAs (top: Mn atoms moved out of the linear chain by local displacements).

The Rietveld analysis is consistent with earlier reports that MnAs takes the MnP-type structure (*Pnma*) at 350 K The orthorhombic distortion is, however, quite weak (see Supporting Information Figures S4 and S5). On further heating, an ideal hexagonal symmetry is regained at around 400 K. For the refined structure, we obtain a Mn–Mn–Mn bond angle of 170.6° at 350 K. This distortion is significantly higher than found from the PDF analysis of (hexagonal) MnAs at 298 K. We postulate that the short Mn–Mn bond distances along the hexagonal *c*-axis are a major cause for the displacements in the MnP-type phase. Upon cooling from the high-temperature hexagonal state, strain resulting from the contraction of the unit cell, and hence of the *c*-axis, is counterbalanced by displacing the Mn atoms out of the chain, thereby obtaining a zig-zag configuration (Figure 5), i.e., turning to the orthorhombic MnP-type structure via a displacive second-order transition. Our PDF data suggest that a similar, but much less extensive, situation exists for the Mn–Mn chain in ferromagnetic MnAs at 298 K. Hence, to reduce repulsions along [001], local displacements occur in line with what is expected for an orthorhombic variant of the structure. In other words, certain features characteristic of the lower-symmetry, high-temperature paramagnetic structure are locally already in place for the higher-symmetry ferromagnetic state at 298 K.

The presence of such a local distortion is consistent with theoretical studies. Bean and Rodbell proposed that the magnetostructural phase transition in MnAs could occur in a compressible material with a strain-dependent exchange energy, critical Mn–Mn separation, and ferromagnetic order inducing an exchange-strictive expansion in the basal plane.^11,40^ Rungger and Sanvito confirmed not only the dependence of ferromagnetic exchange couplings on volume but also that the exchange is dependent on any orthorhombic distortion toward the MnP structure.^23^ The recent computational study by Seshadri et al. proposes the existence of local structural deviations in the hexagonal and orthorhombic phases and finds that the distortions are due to magnetic fluctuations.^36^ Due to the facile presence of local distortions in MnAs, any chemical modification made by means of solid solutions of smaller-sized atoms will generate a chemical pressure that stabilizes the orthorhombic structure for cation- (V, Cr, Fe, Co, Ni, Mo) and/or anion (P)-substituted samples. It is reported that efforts to enhance the magnetocaloric property at the FOMT have not been fruitful since the substituted samples always crystallize with the orthorhombic structure resulting in a significantly reduced magnetic response compared to pure MnAs. This change in magnetic properties is not just a dilution effect for the magnetic sublattice; it is a genuine result of the Mn–Mn displacements and lower-spin state of the orthorhombic phase.

Upon very small substitution levels at the Mn-site, less than around 3 atom %, the FOMT is retained; however, the transition temperature is lowered from that of MnAs (318 K).^41^ It is this composition range that still may hold potential for optimized substitutions that can enhance magnetocaloric properties. However, so far, experimental data also indicate a lowering in the saturation moment, larger than expected based on pure dilution. This suggests the possibility that although globally just below *T*_C_ MnAs and its slightly substituted derivatives have transitioned into the hexagonal NiAs structure, it may locally exhibit displacements reminiscent of a MnP-type like structure that locally reduces the Mn spin state and the ferromagnetic exchange coupling. Hence, local distortions become important for the global magnetic properties of the material.

## Conclusions

Big box modeling of PDF data in combination with small box modeling, as well as EXAFS and PXRD, unequivocally establishes that MnAs at 298 K exhibits local orthorhombic distortions while the average structure remains of hexagonal symmetry. We ascribe the local distortions as a consequence of strong magnetic coupling between high-spin Mn atoms as well as a means to compensate for Mn–Mn bonds that are too short upon cooling of the high-temperature phase. This study provides a rationale for why the orthorhombic structure with a reduced magnetic response is stabilized in MnAs substituted at the Mn and/or As sites. It is important to note that these subtle behaviors of MnAs have been unlocked owing to advanced characterization tools. Insight into the mechanism for structural distortions, spin states, and magnetic interactions is a key for bringing the remaining mysteries of MnAs to light, as a basis for making realistic applications of this excellent material within magnetocalorics. This study demonstrates the strength of combining total scattering with complementary techniques to unravel how local disorder and displacement contribute to structure–property correlations. This combination of tools has major potential to help understand the implications of magnetic interactions on the local atomic structure.

## Supporting Information Available

PDF data fitted with the hexagonal structure in the *r-*range 2–40 Å (Figure S1); Rietveld refinement parameter for MnAs at room temperature (Table S1); Mn EXAFS data fitted with the model generated from orthorhombic and hexagonal structures (Figure S2); atomic parameters at room temperature used in the Rietveld refinement (Table S2); refined anisotropic parameters *U*_aniso_(Å^2^) during the Rietveld refinement (Table S3); refinement parameters after PDF refinement (Table S4); refined atomic coordinates of the orthorhombic structure after PDF refinement (Table S5); atomic coordinates of the hexagonal structure during PDF refinement (Table S6); refined parameters for EXAFS fitting (Table S7); partial functions contributing to Mn–As, Mn–Mn, and As–As to the overall PDF (Figure S3); anisotropic thermal parameter after RMCProfile refinement on MnAs (Table S8); Rietveld refinement of diffraction data collected at 350 K against the orthorhombic structure (Figure S4); Rietveld refined atomic parameters at 350 K (Table S9); Rietveld refinement parameter for 350 K (Table S10); variable-temperature PXRD data for MnAs; peaks (marked with *) are due to the orthorhombic distortion; a zoomed view is presented to highlight weak peaks in the 350 K data (Figure S5); and *x*,*y*,*z* components of a vector describing the rotation axis and magnitude of MnAs_6_ octahedra (Figure S6) (PDF)
