# Supplementary material for: Local Symmetry Deviation from the Average Structure of MnAs Revealed by Pair Distribution Function
Source: Inorg Chem. 2024 Aug 8;63(33):15503–9. doi: 10.1021/acs.inorgchem.4c02667 (PMC11337163; doi:10.1021/acs.inorgchem.4c02667)
Supplement: Supplementary file 1 — ic4c02667_si_001.pdf [file ic4c02667_si_001.pdf]

Supporting information for

Local symmetry deviation from the average structure of MnAs revealed by Pair Distribution Function

Dipankar Saha[a], Wojciech Sławiński*[b], Susmit Kumar[a,c] and Helmer Fjellvåg*[a]*

affiliation

[a] Dr. Dipankar Saha*, Dr. Susmit Kumar, Prof. Helmer Fjellvåg*

Center for Materials Science and Nanotechnology, Department of Chemistry, University of Oslo,

P.O. Box 1033, Blindern, N-0315 Oslo, Norway

E-mail: dipankar.saha@kjemi.uio.no

E-mail: helmer.fjellvag@kjemi.uio.no

[b] Dr. Wojciech Sławiński*

Faculty of Chemistry

University of Warsaw

Ludwika Pasteura 1, 02-093 Warszawa, Poland.

E-mail: wslawinski@chem.uw.edu.pl

[c] Dr. Susmit Kumar

Currently at

Justervesenet - Norwegian Metrology Service (JV), National Laboratory

Fetveien 99, 2007 Kjeller, Norway.
E-mail: sku@justervesenet.no

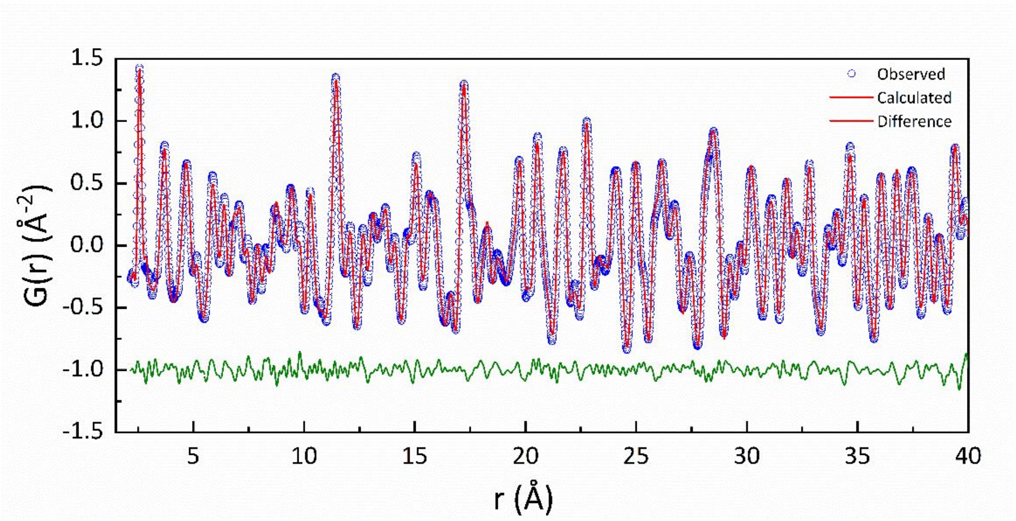

Figure S1: PDF data fitted with hexagonal structure in the r range 2-40 Å.

Table S1: Rietveld refinement parameter for MnAs at Room Temperature.

Space group	$P6_3/mmc$
a(Å)	3.72132(3)
c(Å)	5.70548(2)
Volume(Å^3)	68.42 (12)
Rp(%)	2.87
Rwp(%)	4.52
DWD	1.56
CHI^2	1.05

Table S2: Atomic parameters at room temperature used in Rietveld Refinement.

Atom	Wykoff position	S.O.F	x	y	z	U_{iso}(Å^2)
Mn	2*a*	1	0	0	0	0.006(4)
As	2*c*	1	0.333	0.6667	0.25	0.005(2)

Table S3. Refined anisotropic parameters U_{aniso}(Å^2) during Rietveld Refinement.

Atoms	U11	U22	U33	U12	U13	U23
Mn	0.0076(2)	0.0076(3)	0.0026(1)	0.0038(2)	0	0
As	0.0043(1)	0.0043(1)	0.0079(2)	0.0022(1)	0	0

Table S4: Refinement parameters after PDF refinement.

	Orthorhombic	Hexagonal
Data range	2.1 – 5.5 Å	2.1 – 5.5 Å
Number of data points	332	332
Number of Refined parameters	17	8
Space group	*Pnma*	$P6_3/mmc$
Qdamp	0.0038	0.0038
R_w	6.24	11.40
a (Å)	5.7258(2)	3.7326 (3)
b (Å)	3.7813(5)	3.7326 (3)
c (Å)	6.3367(3)	5.6855 (2)
delta2	4.24	4.16

Table S5: Refined Atomic coordinates of orthorhombic structure after PDF refinement.

Atom	x	y	z	
Mn	0.495(3)	0.25	0.277(2)	0.0139(3)
As	0.274(1)	0.25	0.918(1)	0.0102(2)

Table S6: Atomic coordinates of Hexagonal structure during PDF refinement.

Atom	Wykoff position	S.O.F	x	y	z	U_{iso}(Å^2)
Mn	2*a*	1	0	0	0	0.007(5)
As	2*c*	1	0.333	0.6667	0.25	0.006(1)

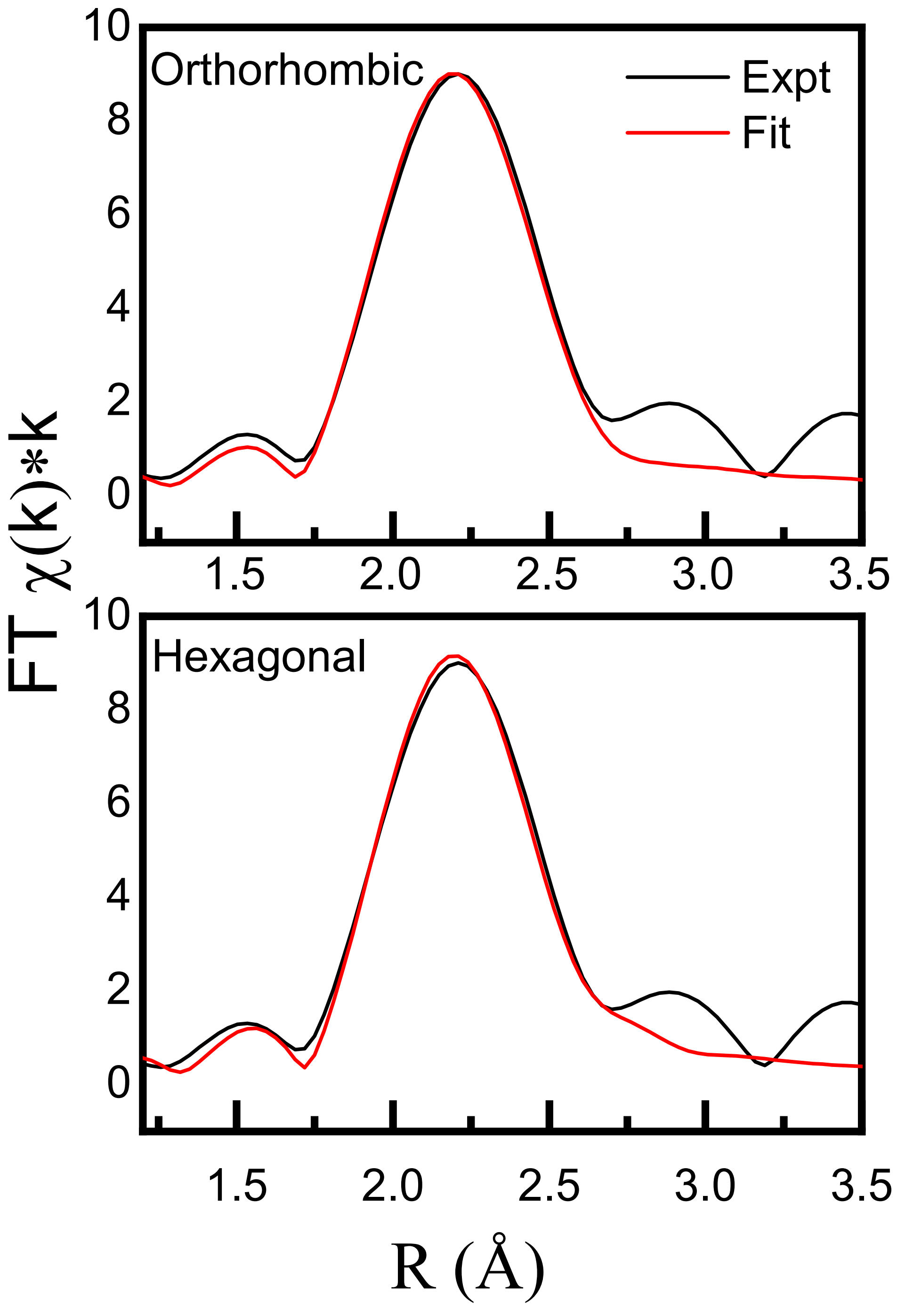

Figure S2: Mn EXAFS data fitted with of model generated from orthorhombic and hexagonal structure.

Table S7: Refined parameters for EXAFS fitting.

	**Path**	**Coordination Number**	**R(Å)**	**ΔE_0 (eV)**	**σ^2(Å^2)**
Hexagonal	**Mn-As**	6	2.61(3)	-5.45(2.1)	0.006(0.0002)
Orthorhombic	**Mn-As**	6	2.53(2)	-5.55(1.9)	0.001(0.0002)

	Hexagonal	Orthorhombic
Independent points	10.55	10.55
Number of variables	4	6
Reduced chi-square	42.79	23.34
R-factor	0.0132	0.007

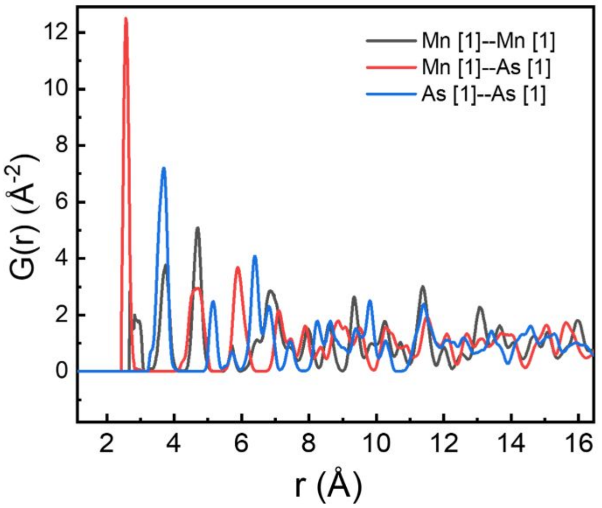

Figure S3: Partial functions contributing of Mn-As, Mn-Mn and As-As to overall PDF are shown.

Anisotropic thermal Parameter from RMCProfile refinement

Primely, we have to point out that the Reversed Monte Carlo method implemented in RMCProfile7 program does not use iso/anisotropic temperature parameters and its representation of U_{ij}. In contrast, in order to model atomic displacement from the average position, in a big box model, all atoms are to be displaced from its average position. Also, no symmetry restrictions are applied (the refinement is run in P1) and so there is no constrain/restrain on those displacements. This is why U_{ij} parameters are not typically extracted from the big box configuration. In many cases atoms displaced from the average do not form ellipsoidal shape and so, the use of U_{ij} representation is not relevant. However, in the case of MnAs, we can see that the final atom cloud (back projected into a single, small unit cell) has very similar shape and size as the thermal ellipsoid obtained from Rietveld refinement (small box or Bragg diffraction refinement). For the purpose of this comment, we have calculated U_{ij} parameters for each of two Mn and two As positions in the full unit cell. Due to the fact that there were no symmetry reinstructions on each site, all non-diagonal U_{ij} components were also calculated.

Table S8: Anisotropic Thermal Parameter after RMCProfile refinement on MnAs.

Atoms	U11	U22	U33	U12	U13	U23
Mn at (0,0,0)	0.0107	0.0107	0.0040	0.0053	0.0001	0.0000
Mn at (0,0,1/2)	0.0108	0.0109	0.0040	0.0056	0.0000	0.0000
As1 at (1/3,2/3,1/4)	0.0075	0.0076	0.0057	0.0038	0.0000	0.0000
As2 at (2/3,1/3,3/4)	0.0075	0.0076	0.0057	0.0038	0.0000	0.0000

As one can see from the table above, for both Mn and both As sites, all U_{ij} values are nearly identical. This means that there is no difference between those crystallographically equivalent positions. Also one can observe that all U_{13} and U_{23} parameters are equal to zero which is fully consistent with the symmetry of the site in hexagonal cell.

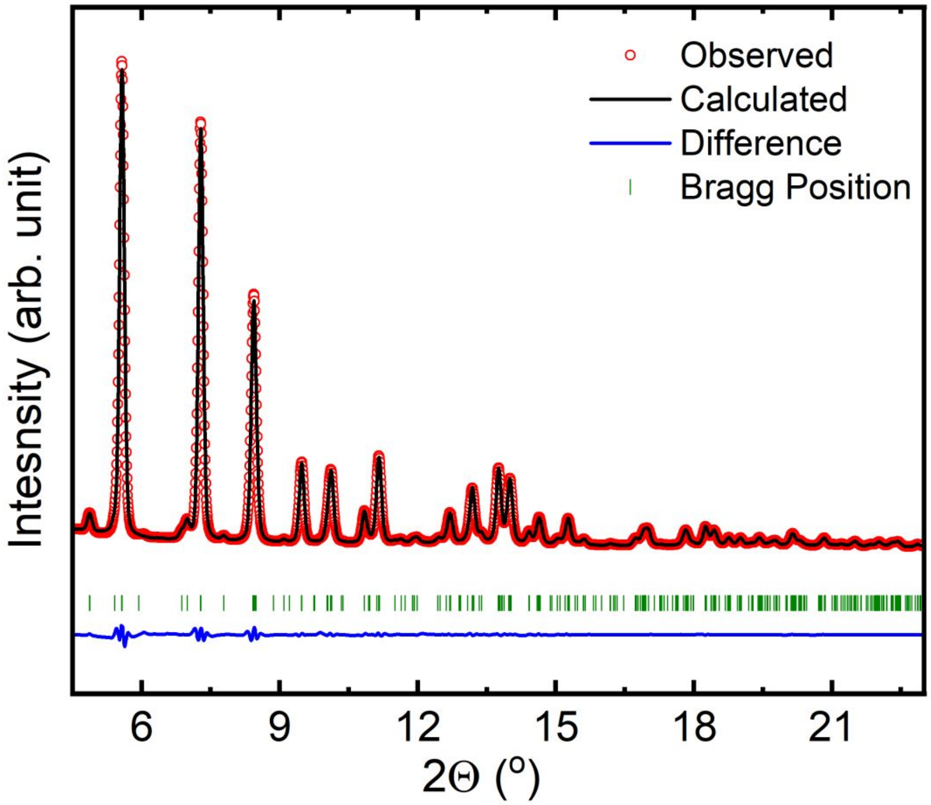

Figure S4: Rietveld refinement of diffraction data collected at 350 K against orthorhombic structure.

Table S9. Rietveld Refined Atomic parameters at 350 K.

Atom		Wykf	S.O:F	x	y	z	U_{iso}
Mn		4c	1	0.0033(2)	1/4	0.2315(1)	0.0153(4)
As		4c	1	0.2313(4)	1/4	0.5824(2)	0.0125(5)

Table S10: Rietveld refinement Parameter for 350 K.

Space group	*Pnma*
a(Å)	5.7318(1)
b(Å)	3.6749(3)
c(Å)	6.3864(5)
Volume(Å^3)	134.52(2)
Rp(%)	2.96
Rwp(%)	6.34
DWD	1.34
CHI^2	0.97

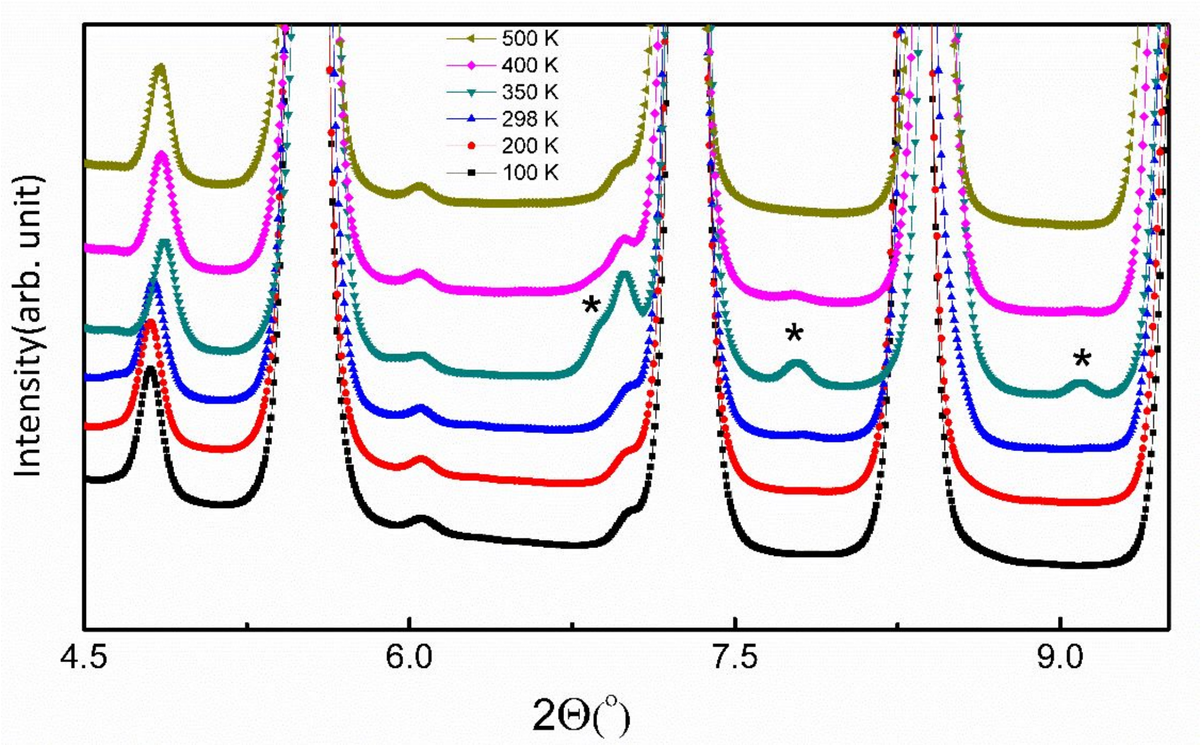

Figure S5: Variable temperature PXRD data for MnAs. Peaks (marked with *) are due to orthorhombic distortion. Zoomed view is presented to highlight weak peaks in the 350 K data.

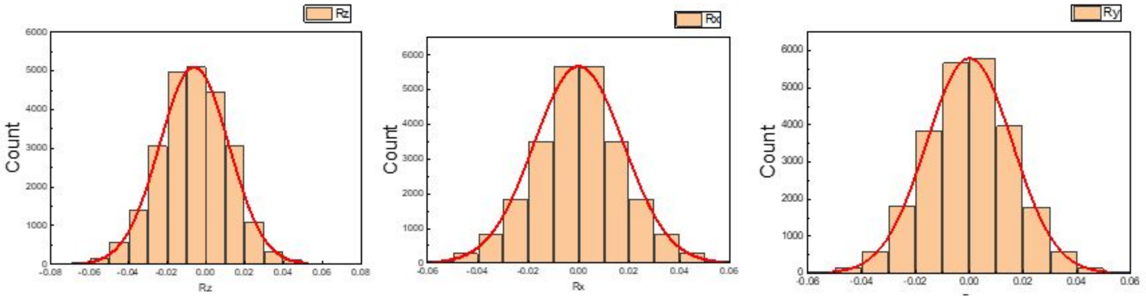

Figure S6: x,y,z components of a vector describing the rotation axis and magnitude of $MnAs_6$ octahedra.
